# Pediatric Acute Myeloid Leukemia Post Cytotoxic Therapy—Retrospective Analysis of the Patients Treated in Poland from 2005 to 2022

**DOI:** 10.3390/cancers15030734

**Published:** 2023-01-25

**Authors:** Małgorzata Czogała, Wojciech Czogała, Katarzyna Pawińska-Wąsikowska, Teofila Książek, Karolina Bukowska-Strakova, Barbara Sikorska-Fic, Paweł Łaguna, Jolanta Skalska-Sadowska, Jacek Wachowiak, Anna Rodziewicz-Konarska, Małgorzata Moj-Hackemer, Krzysztof Kałwak, Katarzyna Muszyńska-Rosłan, Maryna Krawczuk-Rybak, Anna Fałkowska, Katarzyna Drabko, Marta Kozłowska, Ninela Irga-Jaworska, Katarzyna Bobeff, Wojciech Młynarski, Renata Tomaszewska, Tomasz Szczepański, Agnieszka Chodała-Grzywacz, Grażyna Karolczyk, Katarzyna Mycko, Wanda Badowska, Karolina Zielezińska, Tomasz Urasiński, Natalia Bartoszewicz, Jan Styczyński, Walentyna Balwierz, Szymon Skoczeń

**Affiliations:** 1 Department of Pediatric Oncology and Hematology, Institute of Pediatrics, Jagiellonian University Medical College, 30-663 Krakow, Poland; 2 Department of Pediatric Oncology and Hematology, University Children Hospital, 30-683 Krakow, Poland; 3 Department of Medical Genetics, Institute of Pediatrics, Jagiellonian University Medical College, 30-663 Krakow, Poland; 4 Department of Clinical Immunology, Institute of Pediatrics, Jagiellonian University Medical College, 30-663 Krakow, Poland; 5 Department of Pediatrics, Oncology, Hematology and Transplantology, Medical University of Warsaw, 02-091 Warszawa, Poland; 6 Department of Pediatric Oncology, Hematology and Transplantology, Poznan University of Medical Sciences, 60-572 Poznan, Poland; 7 Department of Bone Marrow Transplantation, Pediatric Oncology and Hematology, Medical University of Wroclaw, 50-556 Wroclaw, Poland; 8 Department of Pediatric Oncology and Hematology, Medical University of Bialystok, 15-089 Bialystok, Poland; 9 Department of Pediatric Hematology, Oncology and Transplantology, 20-093 Lublin, Poland; 10 Department of Pediatrics, Hematology and Oncology, Medical University of Gdansk, 80-210 Gdansk, Poland; 11 Department of Pediatrics, Oncology and Hematology, Medical University of Lodz, 91-738 Lodz, Poland; 12 Department of Pediatric Hematology and Oncology, Zabrze, Medical University of Silesia, 40-055 Katowice, Poland; 13 Department of Pediatric Hematology and Oncology, Regional Polyclinic Hospital in Kielce, 25-736 Kielce, Poland; 14 Department of Pediatrics and Hematology and Oncology, Province Children’s Hospital, 10-561 Olsztyn, Poland; 15 Department of Paediatrics, Hemato-Oncology and Gastroenterology, Pomeranian Medical University in Szczecin, 71-252 Szczecin, Poland; 16 Department of Pediatric Hematology and Oncology, Collegium Medicum, Nicolaus Copernicus University Torun, Bydgoszcz, 85-094 Bydgoszcz, Poland

**Keywords:** acute myeloid leukemia post cytotoxic therapy, children, secondary malignancy, therapy-related

## Abstract

**Simple Summary:**

Acute myeloid leukemia post cytotoxic therapy (AML-pCT) is a rare complication of cancer treatment in childhood. We retrospectively analyzed the data of 40 children with AML-pCT, treated from 2005 to 2020. The most common primary malignancies were acute lymphoblastic leukemia and brain tumors. The probabilities of overall (OS), event-free (EFS), and relapse-free survival (RFS) in whole cohort were 0.49 ± 0.08, 0.43 ± 0.08, and 0.64 ± 0.10, respectively. Significant improvements in outcomes were observed in patients treated from 2015 to 2022 (two induction cycles followed by stem cell transplantation—SCT in 69% of patients) compared to the period 2005–2014 (four induction cycles followed by SCT in 49% of patients). The probabilities of EFS and RFS increased from 0.30 ± 0.10 and 0.46 ± 0.11 to 0.67 ± 0.12 and 1.0, respectively. The poorest outcome was found in AML post brain tumor, mainly due to toxic deaths. Treatment results in the group of patients with AML-pCT treated from 2015–2022 were comparable to outcomes in de novo AML.

**Abstract:**

Acute P./myeloid leukemia post cytotoxic therapy (AML-pCT) is rare complication of cancer treatment in childhood. The objective of the study was to identify clinical characteristics and provide an analysis of the outcomes in pediatric AML-pCT. We retrospectively analyzed the data of 40 children with AML-pCT, treated from 2005 to 2020 within the Polish Pediatric Leukemia and Lymphoma Study Group. The most common primary malignancies were acute lymphoblastic leukemia (32.5%) and brain tumors (20%). The median latency period was 2.9 years (range: 0.7–12.9). Probabilities of overall (OS), event-free (EFS), and relapse-free survival (RFS) in the whole cohort were 0.49 ± 0.08, 0.43 ± 0.08, and 0.64 ± 0.10, respectively. Significant improvements in outcomes were observed in patients treated from 2015–2022 (two induction cycles followed by stem cell transplantation—SCT in 69% of patients) compared to 2005–2014 (four induction cycles followed by SCT in 49% of patients). The probability of EFS increased from 0.30 ± 0.10 to 0.67 ± 0.12 (*p* = 0.07) and RFS increased from 0.46 ± 0.11 to 1.0 (*p* = 0.01). The poorest outcome (OS and EFS 0.25 ± 0.20) was in AML post brain tumor, mainly due to deaths from toxicities. To conclude, treatment results achieved in patients with AML-pCT treated from 2015–2022, with two induction cycles followed by immediate SCT, were better than those reported by other authors, and comparable to the results in de novo AML.

## 1. Introduction

Secondary malignancies are a rare complication of cancer treatment in childhood. Acute myeloid leukemia post cytotoxic therapy (AML-pCT), formerly known as therapy-related AML, is the most common secondary malignancy in childhood [1]. The latest WHO classification of myeloid neoplasms (5th edition, 2022) distinguishes a category of myeloid neoplasms as post cytotoxic, including AML, MDS, and MDS/MPN, arising in patients exposed to cytotoxic (DNA-damaging) therapy for an unrelated condition [2]. The incidence of MDS and AML-pCT in children after therapy for cancers is about 0.5–1% [3,4]. It occurs especially after exposure to alkylating agents, topoisomerase II inhibitors, or radiation therapy [2,5,6]. The most frequent primary malignancies include acute lymphoblastic leukemia, osteosarcoma, Ewing sarcoma, Hodgkin and non-Hodgkin lymphomas, neuroblastoma, rhabdomyosarcoma, and brain tumors [3,6,7,8].

The majority of AML-pCT in adults is associated with TP53 mutations [2]. However, the somatic and germline genomic alterations that drive AML-pCT in children are not well described yet. The study conducted by Schwartz et al. revealed that Ras/MAPK pathway mutations, alterations in RUNX1 or TP53, and KMT2A rearrangements were the most frequently associated with pediatric AML-pCT [6]. 

The prognosis in AML-pCT is worse compared to de novo AML with a 5-year OS of 20–55% [7,8,9]. Therapy should take into account both the cumulative cytostatic dose (especially anthracyclines) and the often already impaired regenerative capacity of the bone marrow.

Here, we present a retrospective analysis of the patients with AML-pCT registered from 2005 to 2022 in the nationwide AML database of the Polish Pediatric Leukemia and Lymphoma Study Group (PPLLSG). The objective of the study was to determine the incidence of AML-pCT and describe the clinical characteristics and outcome of AML-pCT in children.

## 2. Materials and Methods

There were 823 children with AML registered from January 2005 to June 2022 in the nationwide AML database of the PPLLSG (495 treated according to the AML-BFM 2004 Interim protocol from 2005 to 2014, 186 according to the AML-BFM 2012 Registry from 2015 to 2019, and 142 with the AML-BFM 2019 from 2019 to 2022). In 40 patients from the whole cohort (4.9%), preceding malignancy was reported. Those patients were enrolled to this retrospective study. We analyzed data concerning the diagnosis of underlying cancer, therapy of the primary cancer, family history, time interval between primary diagnosis and AML-pCT diagnosis (latency period), characteristics of AML-pCT (cytogenetics and molecular genetics, FAB classification, white blood cells at diagnosis, CSN, and extramedullary involvement), therapy of AML-pCT, and the treatment outcome. Most patient were treated according to current therapeutic protocols for AML. The AML-BFM 2004 Interim protocol (2005–2014) did not contain special recommendations for patients with AML-pCT, while both the AML-BFM 2012 Registry (2015–2019) and AML-BFM 2019 (2019–2022) recommended treatment with AIE and HAM, or Ida-FLA and FLA (Table 1), depending on the pre-treatment, especially the cumulative anthracycline dose and allogenic stem cell transplantation (allo-SCT), if CR, CRp, or at least no evidence of leukemia (NEL) was achieved. Based on that, two therapeutic periods were distinguished: from 2005 to 2014 and from 2015 to 2022. Complete remission was defined with standard criteria (<5% of blasts in bone marrow aspirate smears, an absolute neutrophil count > 1 × 10^9^/L, platelets 100 × 10^9^/L, and no evidence of extramedullary involvement). Early death was defined as a death before the assessment of response after two inductions (42nd–56th day of therapy). Probabilities of survival were calculated with the Kaplan–Meier method. Overall survival (OS) was calculated from the diagnosis of AML-pCT to death from any cause or the last follow-up. Event-free survival was calculated from the diagnosis of AML-pCT to the first event (relapse, death of any cause, failure to achieve remission, or secondary malignancy) or last follow-up. Failure to achieve morphological remission was considered an event on day 0. Relapse-free survival (RFS) was calculated as the time from the first remission to the relapse. Probabilities of survival were presented as decimal fractions with standard errors (SE). The sub-groups were compared with a log-rank test.

Quantitative variables were compared within subgroups with the Mann–Whitney U test or Kruskal–Wallis test with a post hoc test (for more than 2 variables) and qualitative variables with a chi-square test or Fisher’s exact test.

All statistical analyses were performed with STATISTICA, version 13, TIBCO Software Inc, Palo Alto, CA, USA. 

The study was conducted according to the guidelines of the Declaration of Helsinki, and approved by the Ethics Committee of Jagiellonian University (protocol code: 1072.6120.249.2017, date of approval: 21 December 2017). 

## 3. Results

Forty patients were enrolled onto the retrospective study, including twenty-four patients in the first period (2005–2014) and sixteen in the second period (2015–2022). The patients’ characteristics are summarized in Table 1. There were 21 girls and 19 boys, aged 2.7–18.5 (median 12.8 years). The median age at the primary malignancy diagnosis was 9.4 (range 0.6–15.9). The data concerning the history of cancers in the family were available in 19 patients, and in 8 (42%) of them, cancers were diagnosed in family members.

### 3.1. Primary Cancer

Primary diseases included acute lymphoblastic leukemia (ALL, 13 patients), brain tumors (eight patients: six medulloblastoma—MBL, one low grade glioma—LGG, one embryonal tumor not otherwise specified), neuroblastoma (NBL, four patients), rhabdomyosarcoma (RMS, four patients), osteosarcoma (three patients), retinoblastoma (RBL, two patients), hemophagocytic lymphohistiocytosis (HLH, two patients), synovial sarcoma (one patient), juvenile myelomonocytic leukemia (JMML, one patient), nephroblastoma (one patient), immature teratoma (one patient), acute myeloid leukemia (one patient). In one patient, there were two malignancies preceding AML—synovial sarcoma and osteosarcoma. As a treatment of the first malignancy, all patients received chemotherapy. Fourteen patients also received radiotherapy and four patients received hematopoietic stem cell transplantation (SCT). Most patients were treated with multiagent chemotherapy for the primary disease. Thirty one children (77.5%) received both alkylating agents and topoisomerase II inhibitors, seven patients (17.5%) were treated with alkylating agents without topoisomerase II inhibitors, two children (5%) received only topoisomerase II inhibitors. Details of the chemotherapy used in the treatment of the primary disease are shown in Table 2.

### 3.2. Latency Period

The median latency period was 2.9 years (range: 0.7–12.9 years). No significant difference in latency period was found depending on primary disease, kind of chemotherapy (alkylating agents, topoisomerase II inhibitors or both), or use of radiotherapy or SCT in the therapy preceding AML. The details are presented in Table 3. 

### 3.3. AML-pCT Characteristics

The AML-pCT characteristics are presented in Table 4. The most frequent FAB types were M5, M0, M1, M2, and M4. There was no patient with FAB M3 and M6 in our cohort. Median WBC at diagnosis was 4.5 (range: 1.1–309.8)

A karyotype was available in 27 patients (67.5%). Analysis revealed abnormalities in the karyotype in 23 (85.2%) of those patients. A complex karyotype was found in five children (18.5%). Aneuploidies were revealed in fourteen patients (51.8%), including monosomy 7 in four children (14.8%), monosomy 5 and trisomy 8 together in one of them, monosomy Y in three (11.1%), trisomy 8 in two (7.4%), and trisomy 13, trisomy 11, monosomy 5, monosomy 6, monosomy 8, monosomy 17, and monosomy X in one patient each. Results of molecular diagnostics were available in 30 patients (75%) and revealed KMT2A rearrangements in nine patients (30%), including KMT2A::MLLT3 (four patients, 13.3%), KMT2A::MLLT1 (three patients, 10%), KMT2A::AFF1 (one patient, 3.3%); RUNX1::RUNX1T1 fusion in four children (13.3%), and CBFβ::MYH11 fusion in one patient (3.3%). There was no FLT3 mutation found in our cohort. Results of genetics depending on primary cancer is presented in Table 2.

### 3.4. AML-pCT Treatement

In the first period (2005—2014), 23/24 patients were treated according to standard AML protocols (AML-BFM 2004 Interim). One received Ida-FLA and FLA cycles, eleven children (46%) were transplanted, most of them (9/11) received four therapy cycles before SCT, and two patients had two cycles. In the second period (2015–2022), 14/16 patients were treated with standard chemotherapy (AML-BFM 2012 Registry—7, AML-BFM 2019—6, 1 with AML-BFM 2019 with Gemtuzumab ozogamycin), two children received individualized therapy, eleven patients (69%) were transplanted, most of them (10/11) received two chemotherapy cycles before SCT, and one received four cycles (Table 5). 

### 3.5. AML-pCT Outcome

**Figure 1:**
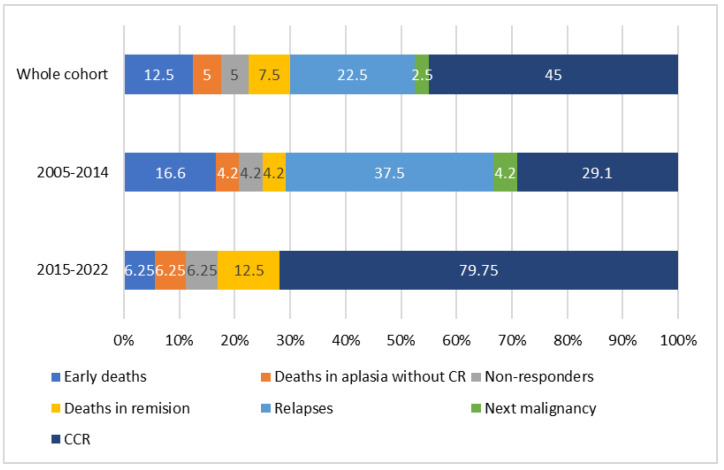
Treatment results in the whole cohort and two periods (2005–2014 and 2015–2022). CR—complete remission, CCR—continuous complete remission.

Thirty one patients (77.5%) achieved complete remission. There were two non-responders (5%), one from each period (2005–2014 and 2015–2022), and both died in the course of the disease progression. One of them was an almost 10-year-old boy, who died 51 months after a NBL diagnosis, with no genetic results available, and 10 months after the AML-pCT diagnosis. The second non-responder was an almost 18-year-old girl with AML-pCT recognized 25 months after an ALL diagnosis. Her karyotype was normal; no fusion gene was found in molecular diagnostics, a WT1 mutation was revealed without an FLT3 mutation. She died 2 months after the AML-pCT diagnosis. Early death occurred in five patients (12.5%), with four from the first period and one from the second, including three patients after brain tumors, one patient after ALL, and one after RMS treatment. In all of them, death was caused by severe infection and multiorgan failure. Two children (5.0%), one from each period, died in course of aplasia without complete remission more than 45 days from the start of therapy. There was one patient after brain tumor therapy and one after RMS who died as a result of severe infections. Three patients (7.5%) died in remission: one in the first period, two in the second, and all of them because of transplantation related toxicities. Of these, there was one patient who died after brain tumor, one after osteosarcoma, and one after immature teratoma. Relapse occurred in nine patients (22.5%), all from the first period. Primary malignancies in that group included ALL (*n* = 5), NBL, RMS, RBL ,and brain tumor (each *n* = 1). The median time from AML-pCT diagnosis to relapse was 10.4 (range: 1.2–27.6) months. There were five patients who relapsed after SCT. Four patients relapsed (44.4%) after achieving second remission, five (55.6%) died of the disease progression, and one patient died in second CR (CRII) because of transplantation related toxicities. Three patients remain in CRII. In one patient (first period), a third malignancy—diffuse large B-cell lymphoma (DLBCL)—occurred 60 months after the AML-pCT diagnosis. The child finally died in the course of the disease. His primary neoplasm was ALL, diagnosed at the age of 14. Treatment results in the whole cohort and two periods are presented on Figure 1. 

**Figure 2:**
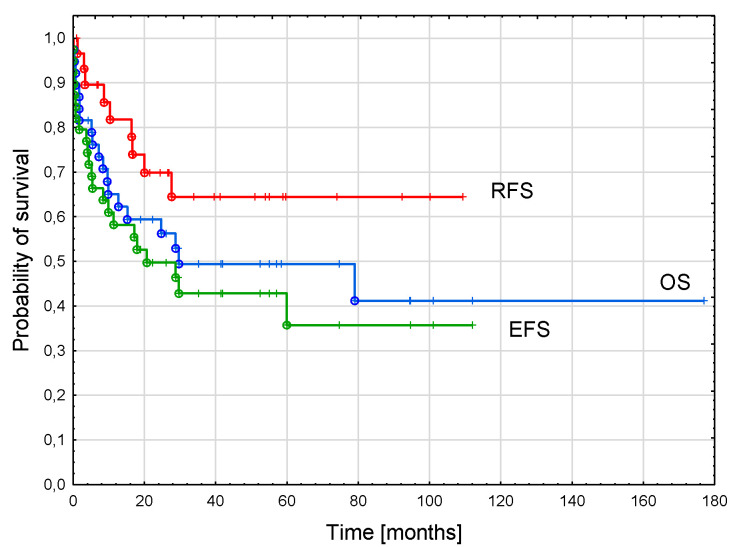
Survival curves of the whole cohort. Three-year overall survival (OS)—0.49 ± 0.08; three-year event-free survival (EFS)—0.43 ± 0.08, three-year relapse-free survival (RFS)—0.64 ± 0.10.

The probability of a 3-year OS in the whole cohort was 0.49 ± 0.08, EFS was 0.43 ± 0.08, and RFS was 0.64 ± 0.01 (Figure 2).

**Figure 3:**
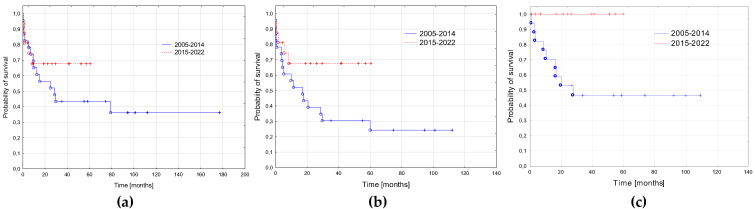
Survival curves in the two periods: 2005–2014 and 2015–2022. (**a**) Probabilities of 3-year overall survival: 2005–2014—0.43 ± 0.1; 2015–2022—0.68 ± 0.12; *p* = 0.35. (**b**) Probabilities of 3-year event free: 2005–2014—0.30 ± 0.1; 2015–2022—0.67 ± 0.12; *p* = 0.07. (**c**) Probabilities of 3-year relapse free survival: 2005–2014—0.46 ± 0.11; 2015–2022—1.0; *p* = 0.01.

The probability of a 3-year RFS was significantly higher in the patients treated in the second period compared to the first period (1.0 vs. 0.46 ± 0.11; *p* = 0.01). There was trend to a better 3-year EFS in the second period (0.67 ± 0.12 vs. 0.30 ± 0.1; *p* = 0.07). The probability of OS did not differ significantly between the groups (0.68 ± 0.12 vs. 0.43 ± 0.1, *p* = 0.35, Figure 3). 

**Figure 4:**
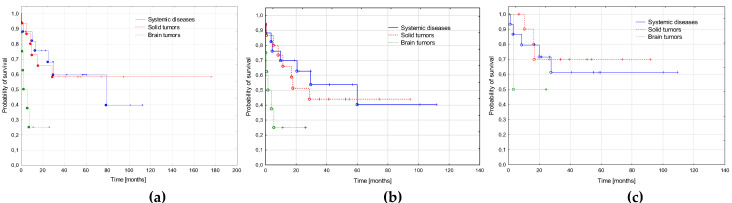
Survival curves depending on the primary diagnosis. Systemic diseases (*n* = 17): acute lymphoblastic leukemia, acute myeloid leukemia, juvenile myelomonocytic leukemia, hemophagocytic lymphohistiocytosis; solid tumors (neuroblastoma, rhabdomyosarcoma, retinoblastoma, nephroblastoma, osteosarcoma, immature teratoma; *n* = 15) and brain tumors (*n* = 8). (**a**) Probabilities of 3-year overall survival: systemic diseases 0.59 ± 0.12, solid tumors 0.58 ± 0.13, brain tumors 0.25 ± 0.2, *p* = 0.023. (**b**) Probabilities of 3-year event free survival: systemic diseases 0.54 ± 0.12, solid tumors 0.44 ± 0.12, brain tumors 0.25 ± 0.2, *p* = 0.079. (**c**) Probabilities of 3-year relapse free survival: systemic diseases 0.61 ± 0.13, solid tumors 0.70 ± 0.15, brain tumors 0.50 ± 0.36, *p* = 0.669.

We compared survival depending on the primary diagnosis. Three groups were distinguished: systemic diseases (ALL, AML, JMML, HLH; *n* = 17), solid tumors (NBL, RMS, RBL, osteosarcoma, teratoma, nephroblastoma; *n* = 15), and brain tumors (*n* = 8). The poorest outcome was observed in AML secondary to brain tumors, with a significantly lower probability of OS (*p* = 0.023) and trend to lower EFS (*p* = 0.079). The probability of RFS did not differ significantly depending on the primary diagnosis. Survival curves are presented in Figure 4 and details of the treatment results in Table 6.

**Figure 5:**
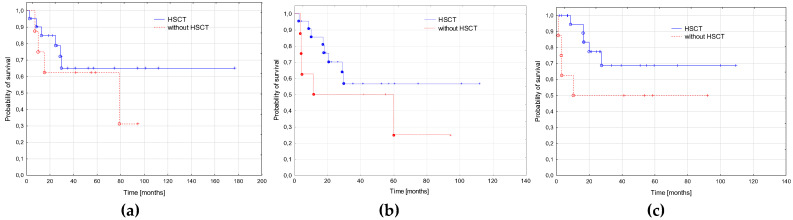
Survival curves depending on the hematopoietic stem cell transplantation (SCT). (**a**) Probabilities of 3-year overall survival: patients treated with SCT (*n* = 22)—0.65 ± 0.09, patients treated without SCT (*n* = 13)—0.62 ± 0.11; *p* = 0.427. (**b**) Probabilities of 3-year event free survival: with SCT—0.57 ± 0.09, without SCT—0.50 ± 0.11; *p* = 0.250. (**c**) Probabilities of 3-year relapse free survival: with SCT—0.69 ± 0.09, without SCT—0.50 ± 0.11; *p* = 0.152.

We also analyzed the influence of SCT on outcome. As all transplantation were performed in CR, we excluded patients who did not achieve CR and those with survival times shorter than 2.6 months (minimal time from diagnosis to SCT in our cohort was 2.6 months). No significant difference in OS, EFS, and RFS was found between patients who underwent SCT and those who did not (Figure 5).

## 4. Discussion

In this retrospective study, we identified 40 patients with AML-pCT in a nationwide registry from a 17.5-year period. Taking into account the average number of newly diagnosed children with malignancies in Poland per year—1100–1200 [10]— the proportion of pediatric patients diagnosed with AML-pCT among all pediatric malignancies was 0.2%. The percentage of AML-pCT among all AML patients was 4.9%. The incidence of myeloid neoplasms post cytotoxic therapy (MN-pCT) including AML-pCT and MDS-pCT reported by other authors was 0.5–1% [3,4,7,11]. Taking into account the proportion of AML-pCT among all MN-pCT 55–75% [6,7,12], it is similar to our study.

Acute lymphoblastic leukemia, osteosarcoma, Ewing sarcoma, Hodgkin and non-Hodgkin lymphomas, NBL, RBL, and brain tumors are the most frequent primary diseases preceding AML-pCT reported by other authors [3,6,7,8]. The most common primary neoplasm in our cohort was ALL (32%), followed by brain tumors (mainly MBL) preceding AML in 20% of our patients. In contrast to other studies, there was no patient with AML post lymphoma in our cohort. Non-malignant disease (HLH treated with etoposide) preceding AML-pCT was diagnosed in two patients in our study. The proportion of the primary malignancies in our study is very similar to that reported by Waack et al. in the study comprising 145 patients with AML-pCT from an AML-BFM study group treated from 1998–2018 [8]. 

The median latency period in our study was 2.9 years, similar to that reported by other authors [7,8,9,12,13]. It seemed that the latency period was longer after the treatment of teratoma, RBL, NBL, AML, JMML, and osteosarcoma (at least 3 years in all the patients), compared to the patients with ALL and HLH as a primary disease (median latency period less than 2 years); however, the differences were not statistically significant, probably because of a low number of patients in subgroups.

Cytogenetic analysis revealed an abnormal karyotype in 85.2% of the children in our cohort. A complex karyotype was found in 18.5% of the patients. Monosomy of chromosome 7 was identified in almost 15% of the children. The most frequent fusion genes identified in our cohort were: KMT2A rearrangements (30%) and RUNX1-RUNX1T1 (13.3%). No FLT3-ITD was identified in our cohort. Similar genetic changes were described by other authors. In the study of Tsurusawa et al., chromosomal abnormalities were detected in 94% of patients, abnormalities of chromosomes 5 and/or 7 in 41%, and KMT2A abnormalities in 31% [13]. Waack et al. reported an abnormal karyotype in 87% of the patients, with a complex karyotype in 10% and KMT2A rearrangements in 47% [8].

It was reported in previous studies that prior exposure to topoisomerase II inhibitors may be associated with translocations involving KMT2A rearrangements and RUNX1 translocations [14,15,16], while monosomy of chromosome 5 or 7 or loss of 5q or 7q is characteristic of alkylating-agent-induced AML [14,15,17]. We did not perform such analysis as most patients in our cohort received both topoisomerase II inhibitors and alkylating drugs. There was no significant difference in the frequency of genetic abnormalities depending on the primary disease in our study. That could be due to small size of the study group.

The treatment of AML-pCT is challenging, taking into account the previous cytotoxic therapy for primary disease, poor hematopoietic reserves, organ dysfunction, and possible colonization with antibiotic-resistant bacteria and fungi as a results of a chronic immunosuppression. In most patients, AML-like induction therapy followed by SCT is used [7,8,12]. In our study, 90% of patients received induction therapy including AIE and HAM cycles, 10% Ida-FLA and FLA cycles, and/or azacytidine and venetoclax. SCT as the first line therapy was performed in 55% of children. It is similar to that reported by other authors. Cho et al. described 13 patients with AML-pCT. All of them received induction therapy including cytarabine and/or idarubicine and 10/13 patients were transplanted [12]. In the study from The MD Anderson Cancer Center, 57% of children with AML-p-CT were treated with cytarabine and an anthracycline, with or without etoposide, and 63% were transplanted [7]. 

Despite these efforts, outcomes in pediatric AML-pCT remains worse compared to de novo AML [3,7,9,13]. However, there has been a significant improvement in AML-pCT treatment outcomes in children over the last 20 years. The studies comprising patients treated before 2010 showed a 3-year OS 15–34% [7,8,18,19]. Better treatment results were observed in children treated in the last decade. The study of the AML-BFM Study Group covering 145 pediatric patients with AML-pCT showed a 5-year OS 28 ± 4%, increasing from 19 ± 5% (*n* = 70 1993–2003) to 34 ± 7% (*n* = 41, 2004–2011), then up to 45 ± 9% (*n* = 34, 2012–2018; p log rank < 0.03) [8]. In the study of Cho et al., 67% of the 12 patients who received AML-type induction therapy achieved complete remission, 25% did not respond, and 8% died during induction therapy [12]. The 5-year OS and EFS of the 13 patients treated with a curative intent were 46.2% and 30.8%, respectively [12]. The outcome in our cohort is even better than that reported by other study groups. In our group, 77.5% of the patients achieved CR and the probabilities of 3-year OS, EFS and RFS were 0.49 ± 0.08; 0.43 ± 0.08 and 0.64 ± 0.10, respectively. Significant improvement in the treatment results was noted in our study as 3-year EFS and RFS increased from 0.30 and 0.46 in the first period (2005–2014) to 0.67 and 1.0 in the second period (2015–2022). The significant difference in RFS could be explained by the shorter follow-up time in the second period. However, all relapses occurred in less than 28 months, while the median follow-up for the second period was 29.5 months. The main difference in the treatment between the two periods was the reduced number of chemotherapy cycles before transplantation in the second period (two cycles instead of four). That could reduce toxicities. Moreover, supportive care in AML patients improved much during last two decades. Data concerning treatment results in the patients with de novo AML treated in a similar period were published by PPLLSG before [20]. It was reported that 3-year OS, EFS and RFS in pediatric patients with de novo AML treated from 2005 to 2015 were 0.67 ± 0.03, 0.53 ± 0.03, and 0.66 ± 0.03, respectively, and from 2015 to 2018: 0.75 ± 0.05, 0.67 ± 0.05, and 0.78 ± 0.05, respectively [20]. While in the first period the outcome in AML-pCT was worse compared to the treatment results in de novo AML, in the second period the treatment results on AML-pCT were similar to those in de novo AML. The percentage of early deaths (12.5%), deaths in aplasia without CR (5%), and deaths in remission (7.5%) in AML-pCT in our study was higher compared to de novo AML in a similar period (early deaths and deaths in remission, 2005–2015: 6% and 5%; 2015–2018: 6% and 1.5%) [20]. It can be explained by bone marrow damage and organ dysfunction as a result of previous cytotoxic therapy for the primary disease. A similar percentage of early deaths in treatment-related AML (14%) was reported in the AML-BFM Study Group [8]. The proportion of non-responders (5%) and relapses (22.5%) in our cohort was similar to that in de novo AML in the report of PPLLSG (2005–2015: non-responders 7%, relapses 31%; 2015–2018: non-responders 7%, relapses 17% [20]). Among all the 19 deaths in our group, 10 were the result of toxicities and 9 of disease progression. That suggest that the main cause of poorer outcomes in AML-pCT compared to the novo AML were deaths from toxicities. Similarly, Cho et al. reported that among 13 patients with AML-pCT treated with a curative intent, five children (38.5%) died because of treatment related toxicities and only two (15.4%) because of disease progression [12]. On the other hand, in the study from MD Anderson Cancer Center, most deaths were caused by disease progression; however, the data were relatively old, concerning patients treated from 1975 to 2007 [7].

We analyzed the influence of primary disease on outcome. We found the poorest outcome in AML secondary to brain tumors compared to systemic malignancies and solid tumors. The main cause of deaths in that group were toxicities. Similarly, the study of Waack et al. revealed that AML following brain tumors as the primary malignancy showed the worst prognosis [8]. Schmiegelow et al. analyzed data on 642 patients with secondary malignancies occurring after treatment for ALL. There were 186 patients with AML-pCT in the analyzed cohort and the 5-year overall survival was 11.2 ± 2.9% for the 125 patients diagnosed before 2000 and 34.1% ± 6.3% for the 61 patients diagnosed after 2000 [21]. In our cohort, there were 13 patients with AML post ALL. They were analyzed together with other systemic malignancies (*n* = 17) and the 3-year OS in that group was 0.46, comparable to the mentioned study. 

Most authors agree that AML-type induction chemotherapy followed by SCT as a post-remission therapy is the most effective way of treating AML-pCT. A significant survival advantage for patients who underwent SCT was reported by many investigators. Brown et al. showed a 5-year survival of 52.4% in patients treated with SCT compared to 5.7% for those who did not have SCT [3]. Schmiegielow et al. found the 5-year survival was 30.3 ± 4.4% for the 119 patients who received transplantation and 11.4 ± 4.0% for the 66 who did not; however, after adjusting for waiting time to transplantation, SCT failed to improve the outcome of secondary myeloid malignancies after ALL [21]. Similarly, in our study, after excluding patients with a survival shorter than minimal time from AML-pCT diagnosis to transplantation, there was no difference in survival between patients who were transplanted and those who were not. Some authors have analyzed the impact of disease status at the time of transplantation on treatment outcomes. In the report of the AML-BFM study group, the poorest outcome was observed in the patients with a persistence of blasts at the time of allo-SCT (10 death/11 patients) [8]. However, in the study of Cho et al., no significant difference in survival was found depending on disease status at the time of transplantation (OS of 80% in CR1/CR2 vs. 50% with persistent disease) [12]. The authors of that study suggested that SCT may be an effective method of treatment even in patients who fail to achieve CR. In our study, all patients who underwent transplantation were in complete remission so we could not perform a similar analysis. 

The major limitation of our study was the small patient numbers, and some missing data, especially concerning cytogenetics and molecular genetics. That precluded robust analyses and the comparison of subgroups. However, most of the previous studies included a similar number of even fewer patients [4,7,9,11,12,13]. Only a few large international studies also including historical data from before 2000 had a larger sample size [8,21]. Considering that AML-pCT is a very rare condition in pediatrics, analysis of even a small cohort seems valuable. In this study, we focused on recent data and found that, nowadays, treatment results in AML-pCT may be similar to those in primary AML. The median observation time in our cohort of almost 54 months (almost 95 months in the first and almost 30 months in the second period) was relatively short compared to other studies. However, taking into account the average time of recurrence of about 10 months, it gives grounds for drawing preliminary conclusions from the obtained results.

Considering that complications were the main cause of treatment failure in our group, it seems that therapy with sufficient efficacy but reduced toxicity, especially in patients heavily pretreated due to primary cancer, may be of key importance for improving outcomes in AML-pCT. The promising results of CPX-351, a fixed 5:1 molar ratio of liposomal cytarabine to daunorubicin, were obtained by Hu et al. The study comprised five pediatric patients with AML-pCT treated with CPX-351. All of them achieved CR, four were transplanted and remained in CR, and one relapsed and died of disease progression before SCT [22]. CPX-351 has been approved in adults and pediatric patients aged ≥ 1 year by the US Food and Drug Administration and in adults by the European Medicines Agency for the treatment of newly diagnosed t-AML or AML with myelodysplasia-related changes. Venetoclax and azacytidine, used mainly in adult patients ineligible for intensive chemotherapy [23,24], may be also an option in AML-pCT, especially for patients with severe toxicities after their primary malignancy treatment. There were some promising studies evaluating azacytidine in pediatric patients with primary AML. A multicenter, open-label, phase II study (NCT02450877) assessed the efficacy of azacytidine in children and young adults with AML in molecular relapse, suggesting that azacytidine may reduce MRD prior to SCT [25]. Another study (NCT01861002) analyzed azacytidine combined with fludarabine plus cytarabine-based chemotherapy in 12 patients with relapsed or refractory (R/R) AML and revealed a 58% rate of complete remission [26]. A phase 3 study evaluating azacytidine versus conventional care regimen in adults with newly diagnosed AML, including 20 patients with therapy-related AML, did not reveal significant differences in OS and CR rate [27]. In the phase 3 VIALE-A study among 105 patients with secondary AML, the addition of venetoclax to azacitidine was associated with improved OS (HR = 0.56 [95 % CI: 0.35, 0.91]) and CR + Cri rate (67 % vs. 23 %, respectively) [28]. 

Research on the use of targeted therapies in pediatric AML may also contribute to improving the outcomes of AML-pCT. Unfortunately, due to the small number of children with AML-pCT and the significant heterogeneity of this disease, it is very difficult to conduct prospective studies in this group. Most studies concern adult patients. Gemtuzumab ozogamicin (GO) is the only antibody–drug conjugate approved by both the Food and Drug Administration and European Medicines Agency in newly diagnosed and refractory CD33-positive AML for patients aged 1 month or older. GO used in combination with standard chemotherapy could be offered for a patient with AML-pCT eligible for intensive chemotherapy. However, the phase 3 EORTC-GIMEMA AML-19 Trial including 73 adult patients with therapy-related or secondary AML showed that first-line monotherapy with low-dose GO, compared with best supportive care, significantly improved OS in older patients who were ineligible for intensive chemotherapy [29]. There was no study on GO in pediatric AML-pCT. Additional agents, not yet approved for AML treatment, are being evaluated in clinical trials for the treatment of AML-pCT in adults. Nivolumab, a PD-1 inhibitor, was tested in a phase 2 study in combination with azacytidine in adults with R/R AML, including 31 patients with secondary AML. The rate of CR or CRi was 33% [30]. Dasatinib, a multikinase inhibitor, was assessed in 89 patients with core-binding factor AML, including 10 patients with t-AML. The overall CR + CRi rate was 94 % and the 4-year OS rate was 52 % [31]. Other agents actually studied in primary AML that could be potentially tested in AML-pCT patients include, among others: eprenetapopt, a small-molecule inhibitor selectively inducing apoptosis in *TP53*-mutated cancer cells, and flotetuzumab, a bispecific antibody binding CD123 and CD3, anti-CD123 CAR-T [14]. Taking into account the high prevalence of KMT2A rearrangements in pediatric AML-pCT, menin inhibitors, blocking the oncogenic function of the KMT2A complex, might be also studied in that group. 

## 5. Conclusions

To conclude, treatment results achieved in patients with AML-pCT treated from 2015–2022 with two induction cycles followed by immediate SCT were better than those reported by other authors, and comparable to outcomes in de novo AML. As the important cause of the treatment failures were toxicities, it seems that novel, less toxic therapeutic agents together with intensive supportive care may play a crucial role in the improvement in the treatment results in AML-pCT in the future.

## Figures and Tables

**Table 1:** The patients’ characteristics.

Period	2005–2014	2015–2022	All Patients
Number of patients	24	16	40
Observation end-point	31/12/2020	31/12/2021	
Median observation time (range) [months]	94.4 (35.2–176.9)	29.5 (4.2–57.1)	53.7 (7.0–176.9)
Gender: male/female	10/14	9/7	19/21
Age at primary cancer diagnosis—median (range) [years]	9.4 (0.5–15.7)	9.3 (0.7–15.9)	9.4 (0.5–15.9)
Age at AML-pCT diagnosis—median (range) [years]	13.0 (2.7–18.2)	12.4 (2.8–18.5)	12.8 (2.7–18.5)
Latency period—median (range) [years]	3.1 (1.5–10.5)	2.7 (0.7–12.9)	2.9 (0.7–12.9)
Primary neoplasm *n* (%)			
ALL	8 (33.3)	5 (31.2)	13 (32.5))
Brain tumors	4 (16.7)	4 (25)	8 (20)
NBL	4 (16.7)	0	4 (10)
RMS	3 (12.5)	1 (6.25)	4 (10)
Osteosarcoma	2 (8.3)	1 (6.25)	3 (7.5))
RBL	1 (4.2)	1 (6.25)	2 (5)
HLH	0	2 (12.5)	2 (5)
JMML	1 (4.2)	0	1 (2.5)
Nephroblastoma	0	1 (6.250	1 (2.5)
Immature teratoma	1 (4.2)	0	1 (2.5)
AML	0	1 (6.25)	1 (2.5)

AML-pCT—acute myeloid leukemia post cytotoxic therapy, ALL—acute lymphoblastic leukemia, NBL—neuroblastoma, RMS—rhabdomyosarcoma, RBL—retinoblastoma, HLH—hemophagocytic lymphohistiocytosis, JMML—juvenile myelomonocytic leukemia, AML—acute myeloid leukemia.

**Table 2:** Alkylating agents and topoisomerase II inhibitors used in the therapy of the primary diseases.

	Alkylating Agents							
	Cyclophosphamide	Ifosfamide	Melphalan	Busulfan	Lomustine	Temozolomide	Cisplatin	Carboplatin
Number of patients (%)	25 (62.5)	14 (35)	3 (7.5)	3 (7.5)	7 (17.5)	1 (2.5)	16 (4)	18 (4.5)
	**Topoisomerase II inhibitors**							
	**Daunorubicin**		**Doxorubicin**		**Idarubicin**		**Etoposide**	
Number of patients (%)	15 (37.5)		19 (47.5)		1 (2.5)		24 (60)	

**Table 3:** Latency period and genetics depending on primary malignancy.

Primary Neoplasm—Number of Patients (%)	Latency Period—Median (Range) [Months]	Genetics —Number of Patients (% of Patients with Available Result)			
		Complex Karyotype	Aneuploidies	KMT2A Rearrangements	CBF Mutations
ALL—13 (32.5)	24.8 (16.2–117.1)	2 (25.0)	3 (37.5)	4 (40)	1 (10)
Brain tumors—8 (20)	30.4 (23.2–54.8)	0	5 (71.4)	2 (33.3)	0
NBL—4 (10)	52.9 (50.6–125.9)	1 (33.3)	1 (33.3)	0	2 (66.6)
RMS—4 (10)	35.7 (22.4–45.7)	1 (50)	1 (50)	0	1 (50)
Osteosarcoma—3 (7.5)	45.6 (42.2–48.9)	1 (50)	1 (50)	1 (33.3)	0
RBL—2 (5)	95.0 (35.1–154.8)	0	1 (50)	0	1 (50)
HLH—2 (5)	20.1 (7.3–32.8)	0	1 (50)	1 (50)	0
JMML—1 (2.5)	52.1	0	0	0	0
Nephroblastoma—1 (2.5)	25.4	0	0	1	0
Immature teratoma—1 (2.5)	121.3	0	1	0	0
AML—1 (2.5)	77.9	0	0	0	0

ALL—acute lymphoblastic leukemia, NBL—neuroblastoma, RMS—rhabdomyosarcoma, RBL—retinoblastoma, HLH—hemophagocytic lymphohistiocytosis, JMML—juvenile myelomonocytic leukemia, AML—acute myeloid leukemia, aneuploidies include: monosomy 5, 6, 7, 8, 17, X, Y, trisomy 8, 11, 13; CBF mutations—core binding factor mutations including RUNX1-RUNX1T1 and CBFβ-MYH11 fusion genes.

**Table 4:** Acute myeloid leukemia post cytotoxic therapy characteristics.

Period	2005–2014	2015–2022	All Patients
Number of patients	24	16	40
FAB types *n* (%)			
M0	5 (20.9)	0	5 (12.5)
M1	0	3 (18.7)	3 (7.5)
M2	4 (16.7)	1 (6.2)	5 (12.5)
M3	0	0	0
M4	4 (16.7)	0	4 (10)
M5	7 (29.2)	3 (18.7)	10 (25)
M6	0	0	0
M7	0	1 (6.2)	1 (2.5)
Non defined	4 (16.7)	8 (50)	12 (30)
WBC at diagnosis—median (range) [10^3^/µL]	5.2 (1.5–120.0)	3.0 (1.1–309.8)	4.5 (1.1–309.8)
Cytogenetics—number of results (%)	16 (66.7)	11 (68.7)	27 (67.5)
Normal karyotype	2 (12.5)	2 (18.2)	4 (14.8)
Complex karyotype	3 (18.7)	2 (18.2)	5 (18.5))
Monosomy 7	2 (12.5)	2 (18.2)	4 (14.8)
Monosomy Y	0	3 (27.3)	3 (11.1)
Trisomy 8	1 (6.2)	1 (9.1)	2 (7.4)
KMT2A rearrangements	2 (12.5)	4 (36.4)	6 (22.2)
t(8;21)(q22;q22)	2 (12.5)	1 (9.1)	3 (11.1)
inv16	1 (6.2)	0	1 (3.7)
Molecular genetics—number of results (%)	14 (87.5)	16 (100)	30 (75)
KMT2A rearrangements	2 (14.3)	7 (43.7)	9 (30)
RUNX1::RUNX1T1 fusion	2 (14.3)	2 (12.5)	4 (13.3)
CBFβ::MYH11 fusion	1 (7.1)	0	1 (3.3)
No fusion genes found	9 (64.3)	7 (43.7)	16 (53.3)

FAB—French–American–British classification, WBC—white blood cells.

**Table 5:** Treatment of acute myeloid leukemia post cytotoxic therapy in the two periods.

Period	2005–2014	2015–2022
Number of patients	24	16
Standard AML therapy—number of treated patients (%)	AML-BFM 2004 Interim—23 (96)	AML-BFM 2012 Registry—7 (44) AML-BFM 2019—6 (38) AML-BFM 2019 + GO (6)
Other—number of treated patients (%)	IdaFLA+ FLA—(4)	IdaFLA + FLA, Venetoclax, Azacitidine—1 (6), Venetoclax, Azacitidine—1 (6)
Number of chemotherapy cycles before SCT—median (range)	4 (2–4)	2 (2–4)
SCT—number of patients (%)	11 (46)	11 (69)

AML—acute myeloid leukemia, SCT—hematopoietic stem cell transplantation, IdaFLA—Idarubicine, Fludarabine, Cytarabine, GO—gemtuzumab ozogamycine.

**Table 6:** Treatment results depending on primary diagnosis.

Primary Diagnosis (N)	Non-Responders	Early Deaths	Deaths in Aplasia	Complete Remission	CCR	Deaths in Remission	Relapses	Deaths after Relapse	II CCR
	N (%)								
ALL (13)	1 (7.7)	1 (7.7)	0	11 (84.6)	6 (46.1)	0	5 (38.5)	4 (30.8)	1 (7.7)
Brain tumors (8)	0	3 (37.5)	1 (12.5)	4 (50)	2 (25)	1 (12.5)	1 (12.5)	1 (12.5)	0
NBL (4)	1 (25)	0	0	3 (75)	2 (50)	0	1 (25)	0	1 (25)
RMS (4)	0	1 (25)	1 (25)	2 (50)	1 (25)	0	1 (25)	1 (25)	0
Osteosarcoma (3)	0	0	0	3 (100)	2 (66.7)	1 (33.3)	0	0	0
RBL (2)	0	0	0	2 (100)	1 (50)	0	1 (50)	0	1 (50)
HLH (2)	0	0	0	2 (100)	2 (100)	0	0	0	0
JMML (1)	0	0	0	1 (100)	1 (100)	0	0	0	0
Nephroblastoma (1)	0	0	0	1 (100)	1 (100)	0	0	0	0
Immature teratoma (1)	0	0	0	1 (100)	0	1 (100)	0	0	0
AML (1)	0	0	0	1 (100)	1 (100)	0	0	0	0

ALL—acute lymphoblastic leukemia, NBL—neuroblastoma, RMS—rhabdomyosarcoma, RBL—retinoblastoma, HLH—hemophagocytic lymphohistiocytosis, JMML—juvenile myelomonocytic leukemia, AML—acute myeloid leukemia, CCR—continuous complete remission.

## Data Availability

The data presented in this study are available on request from the corresponding author. The data are not publicly available due to privacy and ethical restrictions.
